# Ratophobia

**Published:** 1870-04

**Authors:** 


### RATOPHOBIA.

A FEW days since two naughty little children tormented a poor little mouse by pouring oil over it, and setting it on fire; this so enraged the little creature that it bit the finger of one, and fastened itself to the leg of another by digging the teeth deep into the flesh; they both died in a few days.

A man in Jersey City commenced whipping a favorite house dog, holding it up by the tail with one hand with a rod in the other. The enraged animal bit him on the hand, and he died of hydrophobia in a few weeks afterwards.

There are other facts to show that, in an enraged condition of the feelings, a substance escapes from animals which is certain death to other animal natures. Hence it is well to give mad animals as well as mad men, a wide berth.

